# Dietary Inflammatory Index and Blood Pressure Levels in Mexican Adults

**DOI:** 10.3390/nu16183052

**Published:** 2024-09-10

**Authors:** Paola Villaverde, Berenice Rivera-Paredez, Anna D. Argoty-Pantoja, Rafael Velázquez Cruz, Jorge Salmerón

**Affiliations:** 1 Research Center in Policies, Population and Health, School of Medicine, National Autonomous University of Mexico (UNAM), Mexico City 04510, Mexico; paovila@gmail.com (P.V.); argotyanna@gmail.com (A.D.A.-P.); 2 Genomics of Bone Metabolism Laboratory, National Institute of Genomic Medicine (INMEGEN), Mexico City 14610, Mexico; rvelazquez@inmegen.gob.mx

**Keywords:** blood pressure, hypertension, dietary inflammatory index, Mexican population, adults

## Abstract

Background: The relationship between the dietary inflammatory index and blood pressure has been evaluated in European and American populations. This association remains unexplored in Mexico, where outcomes may differ due to the populace’s ancestral heritage and its diverse dietary habits. Methods: We used the Health Workers Cohort Study (2004 to 2018). DII intake was assessed using a food frequency questionnaire. Blood pressure was measured following standardized procedures and techniques. Fixed-effects linear regression and Cox regression models were utilized as the statistical approaches. Results: In the first approach, we observed a positive association between changes in DII intake and changes in both systolic (SBP β: 3.23, 95% CI 1.11, 5.34) and diastolic blood pressure (DBP β: 1.01, 95% CI −0.43, 2.44). When stratified by hypertension, these associations were magnified in participants with hypertension (SBP β: 6.26, 95% CI 2.63, 9.89; DBP β: 1.64, 95% CI −0.73, 4.02). In the second approach, interactions between sex and age categories were explored. Participants in the highest DII category were associated with an increased risk of hypertension, particularly among young women (HR: 3.16, 95% CI 1.19, 8.43). Conclusions: Results suggest that a pro-inflammatory diet is associated with an increase in blood pressure over time among Mexican population.

## 1. Introduction

Inflammation, characterized by the presence of pro-inflammatory cytokines, is a protective response against injury to remove damaged cells and neutralize harmful agents [1]. Chronic inflammation is a biological feature of aging and is increased in obesity [2], with biomarkers such as C-reactive protein (CRP), interleukin-1B/6, and tumor necrosis factor-alpha (TNF-α) increasing with age [3]. Furthermore, obesity can exacerbate inflammation, significantly contributing to hypertension and cardiovascular disease [2].

It is not completely elucidated whether inflammation is a cause or an effect of hypertension. However, inflammatory markers can be elevated in hypertension cases [4,5]. Several prospective trials have associated increased inflammation with a higher risk of incident hypertension [6,7,8]. The possible biological mechanisms underlying these associations is that inflammation downregulates nitric oxide synthase activity, leading to an overproduction of reactive oxygen species, both of which contribute to endothelial dysfunction and oxidative stress, respectively, and are factors that increase blood pressure and cause hypertension [9].

Hypertension affects more than 30% of adults globally and is a significant risk factor for various cardiovascular outcomes and premature death [10]. The prevalence of hypertension among Mexican adults rose by approximately 7% between 2012 and 2018, increasing from 27.2% to 34.1% [11]. Hypertension is a major public health concern in Mexico [11], with high prevalence rates and significant implications for cardiovascular morbidity and mortality. Understanding the dietary factors influencing blood pressure is crucial for developing effective prevention and intervention strategies.

Diet is a crucial environmental and lifestyle factor influencing inflammation. Nutritional components, including micro and macronutrients, specific foods, and overall dietary patterns, are associated with inflammatory responses [12]. For example, saturated fat intake increases certain inflammation markers, while unsaturated fat consumption reduces them [13]. Conversely, being rich in phytocompounds, fruits and vegetables are correlated with an anti-inflammatory effect [14].

Healthy dietary patterns, such as the Mediterranean and Nordic diets, which emphasize high consumption of fruits, vegetables, whole grains, legumes, and fish, with a low intake of red meat and minimally processed foods, have been associated with significant anti-inflammatory potential [15,16]. In contrast, the Western diet, characterized by high energy intake, red and processed meats, sweets, refined cereals, snacks, and sugary drinks, has been linked to increased inflammation markers such as CRP and IL-6 [17].

The dietary inflammatory index (DII), developed by Shivappa et al. [18], is a reliable tool for assessing the inflammatory potential of diets. This index considers the complex interactions within the food matrix and overall dietary patterns. Several studies have examined the association between DII and chronic diseases, including cardiovascular events [19], different types of cancer [20,21,22], metabolic syndrome [23], diabetes [24], obesity [25], memory function [26], and mortality [27].

Most of the research evaluating the relationship between the DII and blood pressure has been conducted using cross-sectional studies, which have the major disadvantage of not being able to establish causality or the direction of relationship between variables. Additionally, most of these studies have been conducted in Asian, European, and American populations [28]. This study explores this relationship in the Mexican population, where results might differ due to the unique ancestry of this population. Mexico is an interesting country to evaluate this relationship, since there is a varied dietary pattern, from the traditional (based on maize, beans, and tortillas) to the modern pattern (soft drinks, fast food, and processed meat). This variability in the Mexican diet provides an ideal environment to evaluate the relation of interest.

Thus, we aimed to assess the DII and blood pressure changes over time in Mexican adults. We hypothesize that a higher DII, indicating a more pro-inflammatory diet, will be associated with increases in both systolic and diastolic blood pressure over time. Additionally, we aim to explore whether these associations are modified by hypertension status and body mass index (BMI). Furthermore, we examined the association between baseline DII and the incidence of hypertension and explored interactions with sex and age categories.

## 2. Materials and Methods

### 2.1. Study Population

The Health Workers Cohort Study (HWCS), which started in 2004, is an ongoing open-cohort study that recruited participants from the Social Security Institute in Mexico (IMSS for its Spanish acronym) and their relatives. The primary objective is to examine the association between lifestyle factors and different health outcomes. Details concerning the study population and design have been reported elsewhere [29].

Briefly, in 2004, participants completed a self-administered questionnaire on sociodemographic characteristics (such as sex, age, education, marital status, among other variables), lifestyle factors (including diet, physical activity, smoking, work environment, and sleeping patterns), and general health status. Following the questionnaire, participants visited the Research Centers at IMSS for a basic clinical examination, including weight and height measurements, total body DEXA, and blood pressure assessment. Additionally, a fasting blood sample was collected for biochemical analysis measures. Similar assessments were conducted during follow-ups in 2010 and 2017.

**Figure 1:**
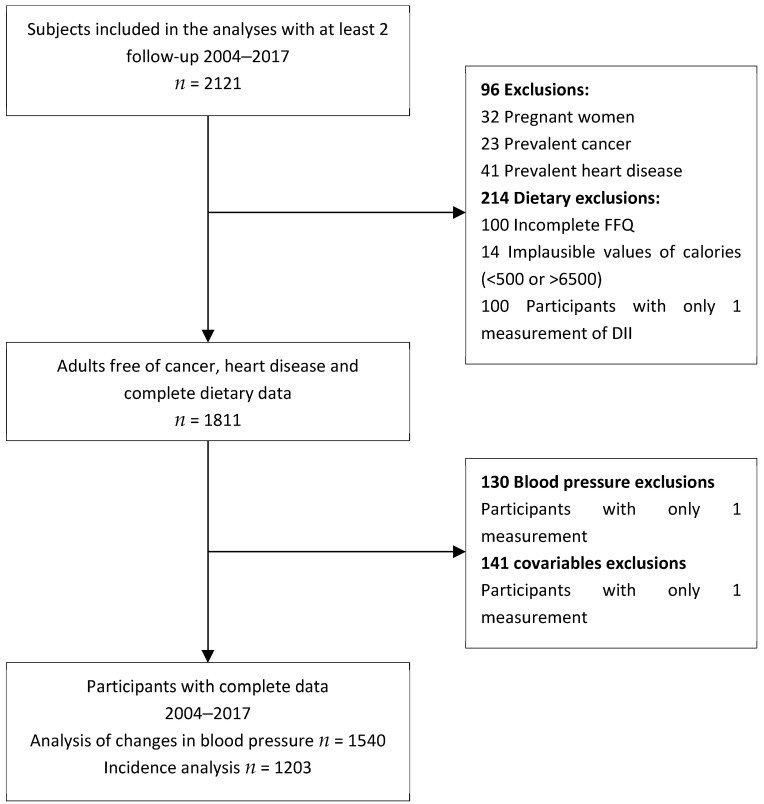
Flow chart of the participants from the Health Workers Cohort Study included in the analytic sample.

For this study, we included participants with at least two follow-ups (*n* = 2121). We excluded those with cancer (endometrial, ovarian, prostate, gastric, colon or rectal, lymphoma, leukemia, lung, or breast), cardiovascular disease (myocardial infarction, angina pectoris, coronary artery surgery), and pregnant women at baseline. Besides, individuals with incomplete data (only one measurement) on hypertension status or covariates were eliminated. Finally, participants were eliminated due to dietary data issues: individuals with an incomplete food frequency questionnaire (FFQ) (<75% of the FFQ), with implausible energy intakes (<500 kcal/day or >6500 kcal/day), and with only one measurement for DII. The final analytical sample included 1540 participants (Figure 1).

### 2.2. Dietary Assessment

Dietary data were collected at baseline using a 116-item self-administered semi-quantitative FFQ validated for the Mexican population in each wave [30]. Participants reported the frequency and portion size of each food or beverage consumed over the previous 12 months. Ten frequency categories were available: never, <1 time/month, 1–3 times/month, 1, 2–4, or 5–6 times/week, and 1, 2–3, 4–5, or 6 or more times/daily. Energy and nutrient intakes were estimated by multiplying the frequency by the content of the specified portion size using the food composition tables compiled by the National Institute of Public Health.

### 2.3. Dietary Inflammatory Index (DII)

We employed methods previously described by Shivappa et al. [18]. Briefly, through a systematic review, they identified studies that evaluated the relationship between inflammatory markers and dietary factors, identifying 45 food parameters associated with 6 inflammatory markers (IL-1β, IL-4, IL-6, IL-10, TNF-α, and CRP). The DII is a score based on these food parameters, ranging from −4.53 to 4.76. A higher DII score indicates a pro-inflammatory, diet while a lower DII score indicates an anti-inflammatory diet.

We estimated the DII by creating a *z*-score for each food parameter in the index. This involved subtracting the individual’s current intake from the global mean provided by the representative world database and then dividing the result by its standard deviation (SD). The *z*-score values were transformed using the empirical cumulative distribution function, then were multiplied by 2 and adjusted by subtracting 1 to obtain a symmetrical distribution centered at 0. The resulting values were then multiplied by the inflammatory score assigned to each food parameter. Finally, the scores for all individual food parameters were summed to calculate the DII [31].

Among the 45 food parameters, 30 were available in the FFQ, including total energy, carbohydrates, proteins, alcohol, fiber, total fats, cholesterol, saturated fat, fatty acids, monounsaturated polyunsaturated fatty acids, trans fat, omega 3, omega 6, caffeine, iron, magnesium, niacin, riboflavin, selenium, thiamine, beta-carotene, zinc, folic acid, onion, and vitamins A, C, D, E, B6 and B12. The following 15 items were not available: rosemary, turmeric, ginger, saffron, garlic, eugenol, pepper, thyme/oregano, anthocyanidins, isoflavones, flavones, flavanols, flavanones, flavan-3-ol, and green/black tea. However, these parameters are not frequently consumed by the Mexican population [31].

### 2.4. Blood Pressure

A medical evaluation was conducted at baseline and in each follow-up period, and data on health status and treatments received were reported. Systolic blood pressure (SBP) and diastolic blood pressure (DBP) were measured twice by trained personnel using an automatic monitor (OMROM HEM-907, OMRON HEALTHCARE, Kyoto, Japan) following standard procedures and techniques [32]. Measurements were taken while participants were seated with their dominant arm supported at heart level, with the first measurement taken after five minutes of rest and the second taken after an additional five-minute interval.

Participants provided information on their medical diagnosis of hypertension, the treatment received, and their year of diagnosis. A self-reported high blood pressure diagnosis was considered hypertension (HTA). The date of HTA diagnosis was determined using two methods: participants who provided the date in the questionnaire had it imputed as the midpoint between their last reported hypertension-free status and the first report of hypertension. At the same time, those diagnosed during the evaluation had their diagnosis date taken from the medical assessment. Participants enrolled in 2004 had two follow-ups on their hypertension status between 2010 and 2017, while those enrolled in 2010 underwent a single follow-up in 2017. According to WHO guidelines, a participant was considered to have hypertension if they reported a physician diagnosis, were receiving antihypertensive treatment, or had an SBP ≥140 mmHg or/and a DBP ≥90 mmHg [33].

### 2.5. Covariates

We considered demographic characteristics such as age, sex, and educational level reported in the questionnaires. Baseline age was categorized as <45 and ≥45 years, since previous studies have reported endocrine changes around 45 years in women that have been associated with cardiovascular risk [34]. Educational level was categorized as primary, high school, graduate or more, and missing. Participants reported their average number of hours of sleep during weekdays and the weekends. Then, we estimated the mean number of hours of sleep during all the whole week. Physical activity (PA) was assessed using a validated questionnaire in a sample with similar characteristics [35,36]. Participants reported weekly recreational PA conducted over the previous year, such as walking, running, etc. We estimated metabolic equivalents (METs) per week by multiplying the hourly average METs for each item based on values from the Compendium of Physical Activities [37]. Smoking status was classified into never, former, or current smokers. A family history of hypertension was reported in the questionnaire (yes/no). Height and weight measures were obtained by trained personnel following standardized procedures [38]. Both measures were used to calculate BMI (kg/m^2^), which was defined as weight divided by the square of height. We used the WHO cut-off points to classify BMI status [39]. Type 2 diabetes (T2D) was defined as a self-reported history of physician-diagnosed T2D, the use of hypoglycemic medication, or fasting glucose with an established cut-off point of ≥126.0 mg/dL [40]. The intake of energy and sodium was obtained from the FFQ.

### 2.6. Statistical Analysis

At baseline, we estimated the changes in continuous variables over a period of five years in the cohort by conducting fixed-effects linear regression models. In these models, each continuous variable was adjusted by the follow-up time divided by 5. Furthermore, we used two modeling approaches to evaluate the relationship of interest. First, we used fixed-effects linear regression to evaluate changes in blood pressure levels, treating the outcome as continuous. In the second approach, we conducted Cox proportional hazards analysis to evaluate the incidence of hypertension. For this analysis, individuals were categorized based on their hypertension status.

#### 2.6.1. Association between Changes in DII and Changes in Blood Pressure

For the first approach (*n* = 1540), we used fixed-effects linear regression models to estimate the longitudinal association between DII intake and blood pressure change over time. This model removes all the time-invariant observed and unobserved characteristics related to DII intake and blood pressure [41]. We categorized individuals according to DII tertiles, with the lowest tertile (T1) representing the most anti-inflammatory diet.

Four models were applied to assess the relation of interest. The first was adjusted for age (years, continuous), and energy intake (kcal/day). Model 2 was adjusted for time-varying covariates that have previously been identified as risk factors for hypertension. Thus, we added to the first model the following variables: physical activity (Met-hour/week, continuous), smoking (never, former, and current), sleep time (hours/d, continuous), and treatment for hypertension (yes/no). Model 3 was similar to model 2, with the only difference being that we additionally adjusted for education (elementary school or less, secondary school or high school, college or higher). Finally, in model 4, we added to model 2 the sodium intake, because this variable was not taken into consideration in the DII.

In addition, several sensitivity analyses were conducted. The analysis was stratified by hypertension status since we did not exclude prevalent cases of hypertension, and thus individuals with this condition could be under treatment modifying the levels of blood pressure. We considered hypertension status at baseline and during the follow-up (2010 and 2017). Further, we excluded participants with diabetes or obesity at baseline, and those who reported weight loss, increased exercise, decreased intake of some caloric foods, and having quit smoking during the follow-up period (2010 and 2017). In both situations, participants could modify their lifestyle style, leading to misclassification in the covariables.

#### 2.6.2. Association between DII and the Incidence of Hypertension

For the second approach (*n* = 1203), we further eliminated prevalence cases of hypertension, and we used Cox proportional hazards regression to estimate hazard ratios (HRs) along with 95% confidence intervals (CIs). The time at entry was the date when the participant received the study questionnaire at the beginning of the follow-up (2004–2010), and the exit time was the date of HTA diagnosis or the censorship date when participants returned the questionnaire in the last follow-up.

We applied four models, the first of which was adjusted for age (years, continuous), sex (men, women), and energy intake (kcal/day, continuous). Model 2, further adjusted for physical activity (Met-hour/week, continuous), smoking (never, former, and current), and sleep time (hours/d, continuous). Model 3 was similar to model 2, with the only difference being that we additionally adjusted for education (elementary school or less, secondary school or high school, college or higher). In the final model 4 we added to model 2, sodium intake.

In this analysis, we conducted the same sensitivity analysis as in the first approach. Additionally, we tested whether BMI influenced the relationship of interest. A previous finding from a French study stratified the relationship by BMI, showing a different effect across categories. Furthermore, all participants in the French study were women and older than the participants in our analysis [42]. For this reason, we additionally stratified by age (<45 years/≥45 years) and sex. Therefore, we stratified the analysis using Cox models. All statistical analyses used Stata 14.0 (Stata Corp, College Station, TX, USA).

## 3. Results

### 3.1. Characteristics of the Study Population

At baseline, among the 1540 participants, the mean age was 45.6 ± 7.3, with the majority being women (73.0%), and the overall prevalence of obesity was 18.6%. In Table 1, we described the changes in continuous variables over a period of five years in the cohort. We observed an increase in age (β = 4.70), SBP (β = 2.37), DBP (β = 2.21), BMI (β = 0.46), and protein intake (β = 7.64). On the other hand, decreases in the sleep time (β = −0.09), physical activity (β = −1.41), energy (β = −180.82), carbohydrates (β = −23.64), total fat (β = −6.10), saturated fat (β = −2.77), sodium (β = −174.43), alcohol intake (β = −0.48 g/day), and fiber intake (β = −1.25 g/day) were found.

### 3.2. Association between Changes in DII and Changes in Blood Pressure

The DII showed a significant positive association with SBP in models adjusted for age and energy, as well as in fully adjusted models. In contrast, no significant association was observed for DBP (Table 2). In model 4, after adjusting for covariates, the average change in SBP associated with progressing from the first to the third tertile was 3.23 mmHg (95% CI 1.11 to 5.34, *p* = 0.003). A significant increase in the average change in SBP was observed in all models when advancing from the first to the second and third tertiles of the DII. For DBP, although an increase in the average change across tertiles was noted, it was not significant in any model.

We performed a sensitivity analysis based on hypertension status at baseline (Table S1). We found a consistent direct relation between DII intake and average changes in blood pressure. For participants with normal blood pressure (Model 4, change from T1 to T3), there were no significant changes in SBP or DBP. However, among hypertensive participants, a significant increase was observed in the average change in SBP across all models (Model 4, beta: 6.26 mmHg, 95% CI 2.63 to 9.89, *p* = 0.001). In contrast, no significant changes were observed in DBP in any model.

In addition, excluding participants who reported lifestyle changes (such as weight loss, increased exercise, reduced intake of certain caloric foods, and smoking cessation) did not alter the results (Table S2).

On average, SBP increased by 4.26 mmHg (95% CI 1.70 to 6.81, *p* = 0.001) and DBP increased by 0.95 mmHg (95% CI −0.82 to 2.73, *p* = 0.283) when comparing T1 to T3, although only the SBP increase was statistically significant. A similar trend was observed when participants with obesity or diabetes were excluded, but only SBP showed a significant increase (3.16 mmHg, 95% CI 0.92 to 5.40, *p* = 0.006) (Table S2).

### 3.3. Association between DII and the Incidence of Hypertension

Increasing DII intake was associated with a non-significant increased risk of hypertension after adjustment for the well-known risk factor for hypertension. In Table 3, in model 4, when consumers in the highest quartile were compared with those in the lowest, participants had a 21% increased risk of hypertension (HR 1.21; 95% CI 0.77 to 1.90 *p*-trend = 0.66). Furthermore, the results were unchanged when participants who reported lifestyle changes, obesity, or diabetes were excluded (Table S3). In addition, no significant changes were observed when we evaluated the potential modifying effect of BMI (Table S4).

In contrast, when the sample was stratified according to sex and age (Table S5), we observed a significant increase in the risk of hypertension in men under 45 years of age (HR 3.17, 95% CI 1.11 to 9.07) in the first model, but after adjustment for confounders, this association was no longer significant. We did not observe this trend for men aged 45 years and older. For women, we observed an increased risk of hypertension only for those under 45 years of age (HR 3.16, 95% CI 1.19 to 8.43).

## 4. Discussion

The results from this prospective study suggest that a pro-inflammatory diet is associated with an increase in systolic blood pressure over time. These results were independent of known risk factors for hypertension such as age, education, BMI, physical activity, smoking, and comorbidities such as diabetes. In addition, when we stratified by hypertension status, the effect was strongest amongst those with hypertension. Using Cox regression models, we observed an increase in the risk of hypertension among participants with a normal weight; however, this was not significant.

We found a significant difference about hypertension status and systolic blood pressure, which leads us to suggest that DII may influence the increase in systolic blood pressure among individuals in our study. A limited number of prospective studies have examined this relationship, and most of these have been conducted in developed countries. In France, the SUVIMAX cohort study [43], which included participants of both sexes, showed that an inflammatory diet at baseline was associated with small increases in blood pressure. However, this increase was significant only for SBP. Similar to our results, the association observed was only significant with SBP. The Australian Longitudinal Study on Women’s Health [44] observed an association between a pro-inflammatory diet with an odds ratio of 1.24 for incident hypertension, when they compared a positive with a negative DII score. Therefore, similar to our results, in both of these studies, a direct association was observed between the DII and changes in blood pressure or hypertension over time, even if they used a different association measure.

A recent trial in older Australian adults [45] found that a Mediterranean diet significantly reduced the dietary inflammatory index (DII) score, but no improvement in cardiometabolic outcomes was observed, despite the reduction in DII. This study supports our observation that although DII may be related to systolic blood pressure (SBP), changes in DII do not always translate into clear clinical improvements. These findings highlight the importance of considering dietary inflammation in the prevention of hypertension and underscore the need for further research on its impact on cardiometabolic health.

Similar to our findings, the French E3N cohort study identified a direct association between DII intake and hypertension risk, specifically among participants within the normal weight range (BMI 18.50–21.49 kg/m^2^) [42]. In our research, we similarly observed an increase in hypertension risk across DII intake quartiles among normal-weight participants; however, this trend did not reach statistical significance. Notably, our study comprised 531 normal-weight participants, in contrast to the E3N study’s 18,566 participants in this category. Consequently, the variance in results might be attributed to the disparity in sample sizes, suggesting a potential lack of statistical power in our study.

For this analysis, we conducted two different statistical approaches to evaluate the relationship of interest, leading to different results. Concerning the model type, fixed effect models control for unobserved heterogeneity by using within-subject variations, which might be more sensitive to detecting changes in continuous variables like blood pressure within individuals over time [46]. On the other hand, Cox regression models evaluate the time-to-event data (time until the event diagnosis) and are more suited for binary outcomes. They may be less sensitive to detecting trends in continuous processes unless the change leads to hypertension [47]. Concerning the outcome, in the fixed effect models, blood pressure was treated as a continuous variable, which captures any incremental changes in blood pressure across the tertiles of DII. This allowed for more sensitivity in detecting subtle changes and trends in blood pressure levels [48]. In contrast, in the Cox regression models, the outcome was dichotomous. This approach may not capture smaller, more gradual increases in blood pressure that did not reach the threshold for hypertension, therefore reducing sensitivity [49,50].

Furthermore, the differences observed between men and women, as well as younger and older age groups, may be attributed to physiological, hormonal, and lifestyle factors. Women are influenced by hormonal variations due to estrogen, which is known to have a protective effect against hypertension. Nonetheless, as women approach menopause, the levels of estrogen decrease, leading to a higher risk of hypertension [51]. In addition, as individuals grow older, there are changes in the structure and function of their vascular system, including arterial stiffness and diminished endothelial function, which increase the risk of hypertension. These changes are more critical in individuals over 45 years old [52]. The protective effect of an anti-inflammatory diet may not be enough to combat the physiological changes typical of age. Consequently, the dietary impact might be more important in younger individuals who have not yet experienced these age-related vascular alterations, making the influence of dietary factors on hypertension risk more apparent [52]. In addition, younger individuals often engage in different lifestyle behaviors, like increased physical activity and distinct dietary habits, compared to older adults. These lifestyle differences can interact with the dietary inflammatory index, potentially influencing the risk of hypertension [53].

The strengths of this study include its population-based prospective design, the wide range of ages covered, and the inclusion of both sexes. We increased the accuracy and precision of dietary measurements by using at least two FFQ measurements, which allowed us to estimate changes in DII. In addition, the use of the fixed effects models can limit the impact of confounding by time-invariant factors or unmeasured characteristics of participants with time-invariant effects. Also, some limitations have to be considered. This is an observational study; therefore, we cannot rule out residual confounding even if we adjusted for the main risk factors for hypertension. Diet was self-reported, and is also associated with measurement error, as it is possible that some participants were misclassified in terms of their exposure. Although our study did not incorporate all food items originally used by Shivappa et al. for DII calculation, such as garlic, known for its anti-inflammatory properties [54], we believe that the absence of these compounds likely had a minimal impact on the accuracy of the DII, as our results are consistent with previous research [42,55,56] and most of these parameters have a low frequency of consumption in the Mexican population [31].

## 5. Conclusions

In conclusion, in this Latin population, we observed a direct association between pro-inflammatory diets and an increase in systolic blood pressure (not generalized to global blood pressure). These findings endorse the notion that individuals vulnerable to hypertension and cardiovascular diseases ought to be motivated to opt for nutritious dietary options like fruits and vegetables, while steering clear of inflammatory foods to mitigate the diet’s pro-inflammatory impact.

## Figures and Tables

**Table 1:** Changes in continuous variables for every 5 years in the Health Workers Cohort Study (*n* = 1540).

Characterisitics	Baseline Mean (SD)	Changes β	(95% CI)	*p*-Value
Age (years)	45.6 (12.9)	4.70	(4.65, 4.75)	<0.001
Systolic Blood pressure (mm Hg)	116.3 (13.1)	2.37	(1.94, 2.80)	<0.001
Diastolic Blood pressure (mm Hg)	71.7 (9.9)	2.21	(1.74, 2.68)	<0.001
Sleep time (hours/d)	7.3 (1.3)	−0.09	(−0.13, −0.05)	<0.001
Physical activity (MET-hour/week)	14.3 (20.9)	−1.41	(−1.98, −0.84)	<0.001
BMI (kg/m^2^)	26.5 (4.4)	0.46	(0.34, 0.58)	<0.001
Dietary variables				
Total energy (kcal/d)	2129 (878)	−180.82	(−205.68, −155.96)	<0.001
Total carbohydrates (g/d)	327.6 (146.6)	−23.64	(−27.96, −19.32)	<0.001
Total Proteins (g/d)	41.2 (26.0)	7.64	(6.78, 8.50)	<0.001
Total fats (g/d)	55.5 (27.4)	−6.10	(−6.86, −5.33)	<0.001
Saturated fats (g/d)	20.3 (10.8)	−2.77	(−3.06, −2.47)	<0.001
Sodium (mg/d)	1939.7 (929.5)	−174.43	(−202.06, −146.81)	<0.001
Alcohol (g/d)	4.5 (10.7)	−0.48	(−0.92, −0.03)	0.035
Fiber (g/d)	29.2 (16.0)	−1.25	(−1.72, −0.78)	<0.001
Dietary inflammatory index	0.58 (2.07)	0.28	(0.23, 0.34)	<0.001

**Table 2:** Blood pressure changes according to the dietary inflammatory index tertiles. The Health Workers Cohort Study, 2004–2017 (*n* = 1540).

	Dietary Inflammatory Index				
Change T1 to T2			Change T1 to T3		
SBP	β	[95% CI]	β	[95% CI]	*p*-Trend
M1	1.55	[−0.11; 3.20]	2.98	[0.92; 5.05]	0.005
M2	1.53	[−0.17; 3.23]	3.10	[0.99; 5.20]	0.004
M3	1.93	[0.05; 3.81]	3.55	[1.20; 5.91]	0.003
M4	1.60	[−0.10; 3.30]	3.23	[1.11; 5.34]	0.003
**DBP**					
M1	0.39	[−0.72; 1.50]	1.00	[−0.38; 2.39]	0.148
M2	0.25	[−0.90; 1.40]	0.96	[−0.47; 2.39]	0.173
M3	0.27	[−0.99; 1.54]	0.94	[−0.64; 2.53]	0.228
M4	0.28	[−0.88; 1.43]	1.01	[−0.43; 2.44]	0.156

SBP: systolic blood pressure; DBP: diastolic blood pressure. T: tertile. β: coefficients. M1: age (years, continuous) and energy intake (kcal/d, continuous). M2: M1 + physical activity (Met-h/week, continuous), smoking (never, former, and current), sleep time (hours/day, continuous) and treatment for hypertension (yes/no). M3: M2 + education (elementary school or less, secondary school or high school, college or higher). M4: M2 + sodium (mg/d, continuous).

**Table 3:** Risk of hypertension according to the dietary inflammatory index quartiles. The Health Workers Cohort Study, 2004–2017 (*n* = 1203).

Quartiles of DII	Q1 (<−1.48)	Q2 (−1.48; −0.03)	Q3 (−0.02; 1.73)	Q4 (>1.73)	
	HR [95% CI]	HR [95% CI]	HR [95% CI]	HR [95% CI]	*p*-Trend
Cases	82	94	85	80	
Person years	9.02	8.44	8.34	8.35	
*n*	301	301	301	300	
M1	Reference	1.26 [0.89; 1.78]	1.17 [0.80; 1.73]	1.22 [0.77; 1.91]	0.62
M2	Reference	1.25 [0.88; 1.76]	1.16 [0.79; 1.71]	1.21 [0.77; 1.90]	0.66
M3	Reference	1.20 [0.85; 1.70]	1.14 [0.77; 1.69]	1.18 [0.74; 1.86]	0.79
M4	Reference	1.25 [0.88; 1.76]	1.17 [0.79; 1.73]	1.21 [0.77; 1.90]	0.66

M1: age (years, continuous), sex (men/women) and energy intake (kcal/d, continuous). M2: M1 + physical activity (Met-h/week, continuous), smoking (never, former, and current) and sleep time (hours/day, continuous). M3: M2 + education (elementary school or less, secondary school or high school, college or higher). M4: M2 + sodium (mg/d, continuous).

## Data Availability

The data is available in this link: https://drive.google.com/drive/u/0/folders/1pUYws_XlWwHhWKDVEJ3laRl_aCv_XmN0 (accessed on 5 September 2024).

## Supplementary Materials

The following supporting information can be downloaded at: https://www.mdpi.com/article/10.3390/nu16183052/s1, Table S1. Blood pressure changes according to the dietary inflammatory index tertiles by hypertension status. The Health Workers Cohort Study, 2004–2017 (*n* = 1540). Table S2. Blood pressure changes according to the dietary inflammatory index excluding participants with lifestyle changes, diabetes, or obesity. The Health Workers Cohort Study, 2004–2017. Table S3. Risk of hypertension according to the dietary inflammatory index quartiles, excluding participants with lifestyle changes, diabetes, or obesity. The Health Workers Cohort Study, 2004–2017. Table S4. Risk of hypertension according to the dietary inflammatory index quartiles stratified by BMI. The Health Workers Cohort Study, 2004–2017. Table S5. Risk of hypertension according to the dietary inflammatory index quartiles stratified by sex and age. The Health Workers Cohort Study, 2004–2017.
