# Supplementary material for: Dietary Inflammatory Index and Blood Pressure Levels in Mexican Adults
Source: Nutrients. 2024 Sep 10;16(18):3052. doi: 10.3390/nu16183052 (PMC11434630; doi:10.3390/nu16183052)
Supplement: Supplementary file 1 [file nutrients-16-03052-s001.zip › nutrients-3181771-supplementary.pdf]

Table S1. Blood pressure changes according to the dietary inflammatory index tertiles by hypertension status. The Health Workers Cohort Study, 2004–2017 (*n*=1540).

	Dietary Inflammatory Index									
	Normal (n=838)					Hypertension (n=702)				
	Change T1 to T2		Change T1 to T3			Change T1 to T2		Change T1 to T3		
**SBP**	**β**	**95%CI**	**β**	**95%CI**	**p-trend**	**β**	**95%CI**	**β**	**95%CI**	**p-trend**
M1	1.05	[-0.58; 2.68]	1.51	[-0.56; 3.58]	0.159	2.92	[0.01; 5.82]	5.73	[2.11; 9.35]	0.002
M2	0.79	[-0.92; 2.49]	1.36	[-0.78; 3.51]	0.216	3.15	[0.25; 6.05]	6.23	[2.61; 9.85]	0.001
M3	1.58	[-0.35; 3.51]	2.20	[-0.24; 4.64]	0.085	3.58	[0.42; 6.73]	6.64	[2.68; 10.61]	0.001
M4	0.89	[-0.83; 2.60]	1.51	[-0.65; 3.67]	0.174	3.16	[0.26; 6.06]	6.26	[2.63; 9.89]	0.001
**DBP**										
M1	0.66	[-0.64; 1.96]	0.95	[-0.70; 2.59]	0.267	0.54	[-1.33; 2.41]	1.56	[-0.77; 3.88]	0.175
M2	0.56	[-0.81; 1.93]	0.86	[-0.86; 2.58]	0.337	0.39	[-1.51; 2.29]	1.58	[-0.79; 3.95]	0.169
M3	0.65	[-0.89; 2.19]	1.03	[-0.93; 2.98]	0.309	0.40	[-1.65; 2.46]	1.50	[-1.08; 4.08]	0.234
M4	0.57	[-0.80; 1.95]	0.87	[-0.86; 2.61]	0.332	0.41	[-1.49; 2.31]	1.64	[-0.73; 4.02]	0.155

SBP: Systolic blood pressure; DBP: Diastolic blood pressure. T: tertile. β: Coefficient
M1: age (years, continuous) and energy intake (kcal/d, continuous)
M2: M1 + physical activity (Met-h/week, continuous), smoking (never, former, and current), sleep time (hours/day, continuous) and treatment for hypertension (yes/no)
M3: M2+ education (elementary school or less, secondary school or high school, college or higher)
M4: M2 + sodium (mg/d, continuous)

Table S2. Blood pressure changes according to the dietary inflammatory excluding participants with lifestyle changes, diabetes or obesity. The Health Workers Cohort Study, 2004–2017.

	Dietary Inflammatory Index									
	Participants without lifestyle changes (*n* =1137)					Participants without obesity or diabetes (*n* =1196)				
	Change T1 to T2		Change T1 to T3			Change T1 to T2		Change T1 to T3		
**SBP**	**β**	**[95% CI]**	**β**	**[95% CI]**	**p-trend**	**β**	**[95% CI]**	**β**	**[95% CI]**	***p*-trend**
M1	2.66	[0.66; 4.67]	4.06	[1.59; 6.54]	0.002	1.96	[0.22; 3.71]	2.92	[0.73; 5.10]	0.010
M2	2.59	[0.53; 4.66]	4.23	[1.68; 6.77]	0.001	1.83	[0.04; 3.63]	2.94	[0.71; 5.18]	0.011
M3	3.09	[0.81; 5.37]	4.51	[1.67; 7.36]	0.003	2.39	[0.40; 4.37]	3.48	[0.99; 5.97]	0.007
M4	2.61	[0.54; 4.68]	4.26	[1.70; 6.81]	0.001	1.96	[0.16; 3.75]	3.16	[0.92; 5.40]	0.006
**DBP**										
M1	0.58	[-0.81; 1.96]	1.02	[-0.69; 2.74]	0.243	0.19	[-1.03; 1.40]	0.59	[-0.93; 2.11]	0.440
M2	0.43	[-1.00; 1.86]	0.99	[-0.77; 2.76]	0.262	0.01	[-1.25; 1.28]	0.51	[-1.06; 2.08]	0.501
M3	0.55	[-1.02; 2.13]	1.08	[-0.89; 3.05]	0.281	0.06	[-1.33; 1.44]	0.51	[-1.23; 2.25]	0.548
M4	0.41	[-1.03; 1.84]	0.95	[-0.82; 2.73]	0.283	0.04	[-1.23; 1.30]	0.56	[-1.02; 2.13]	0.470

SBP: Systolic blood pressure; DBP: Diastolic blood pressure. T: tertile. β: Coefficient
M1: age (years, continuous) and energy intake (kcal/d, continuous)
M2: M1 + physical activity (Met-h/week, continuous), smoking (never, former, and current), sleep time (hours/day, continuous) and treatment for hypertension (yes/no)
M3: M2+ education (elementary school or less, secondary school or high school, college or higher)
M4: M2 + sodium (mg/d, continuous)

Table S3. Risk of hypertension according to the dietary inflammatory index quartiles, excluding participants with lifestyle changes, diabetes or obesity. The Health Workers Cohort Study, 2004–2017.

	Dietary Inflammatory Index							
	**Participants without obesity or diabetes (n=997)**							
	**Q1**	**Q2**		**Q3**		**Q4**		
		**HR**	**[95% CI]**	**HR**	**[95% CI]**	**HR**	**[95% CI]**	**p-trend**
M1	Reference	1.33	[0.89; 2.01]	1.29	[0.82; 2.03]	1.30	[0.77; 2.22]	0.58
M2	Reference	1.32	[0.87; 1.99]	1.28	[0.82; 2.02]	1.30	[0.76; 2.21]	0.61
M3	Reference	1.24	[0.82; 1.87]	1.24	[0.79; 1.95]	1.26	[0.74; 2.14]	0.77
M4	Reference	1.32	[0.88; 2.00]	1.30	[0.83; 2.04]	1.30	[0.77; 2.22]	0.59
	**Participants without lifestyle changes (n=780)**							
M1	Reference	1.15	[0.72; 1.82]	0.97	[0.57; 1.66]	0.86	[0.46; 1.61]	0.66
M2	Reference	1.22	[0.79; 1.89]	1.04	[0.63; 1.72]	0.94	[0.54; 1.64]	0.65
M3	Reference	1.32	[0.84; 2.05]	1.12	[0.67; 1.87]	0.99	[0.56; 1.73]	0.50
M4	Reference	1.20	[0.77; 1.88]	1.02	[0.60; 1.72]	0.91	[0.51; 1.65]	0.64

M1: age (years, continuous), sex(men/women) and energy intake (kcal/d, continuous)
M2: M1 + physical activity (Met-h/week, continuous), smoking (never, former, and current) and sleep time (hours/day, continuous)
M3: M2+ education (elementary school or less, secondary school or high school, college or higher)
M4: M2 + sodium (mg/d, continuous)

Table S4. Risk of hypertension according to the dietary inflammatory index quartiles stratified by BMI. The Health Workers Cohort Study, 2004–2017.

**Quartiles of DII**	**Normal (<25 kg/m^2)** (n = 531) HR [95% CI]	**Overweight (25–29.9 kg/m^2)** (n = 499) HR [95% CI]	**Obesity (> 30 kg/m^2)** (n = 173) HR [95% CI]
Q1	Reference	Reference	Reference
Q2	1.39 [0.70; 2.77]	1.25 [0.77; 2.03]	1.05 [0.49; 2.26]
Q3	1.91 [0.90; 2.04]	1.03 [0.60; 1.76]	1.14 [0.60; 1.76]
Q4	2.05 [0.86; 4.89]	0.89 [0.46; 1.71]	1.34 [0.46; 3.90]
p-trend	0.33	0.52	0.95

Model adjusted for age (years, continuous), sex (men, women), energy intake (kcal/d, continuous), family history of HTA (yes/no), physical activity (Met-h/week, continuous), smoking (never, former, and current), sleep time (hours/day, continuous) and sodium (mg/d, continuous).

Table S5. Risk of hypertension according to the dietary inflammatory index quartiles stratified by sex and age. The Health Workers Cohort Study, 2004–2017.

	**Men**				**Women**			
	**<45 years**		**≥ 45 years**		**<45 years**		**≥ 45 years**	
**Quartiles of DII**	**HR**	**[95% CI]**	**HR**	**[95% CI]**	**HR**	**[95% CI]**	**HR**	**[95% CI]**
Model 1								
Q1	Reference		Reference		Reference		Reference	
Q2	1.16	[0.37; 3.57]	1.34	[0.63; 2.84]	1.76	[0.91; 3.42]	1.06	[0.71; 1.59]
Q3	2.05	[0.72; 5.83	1.33	[0.64; 2.79]	1.18	[0.57; 2.43]	1.11	[0.74; 1.66]
Q4	3.17	[1.11; 9.07]	0.96	[0.44; 2.12]	1.72	[0.86; 3.42]	0.69	[0.45; 1.07]
Model 4								
Q1	Reference		Reference		Reference		Reference	
Q2	0.65	[0.18; 2.31]	0.88	[0.34; 2.27]	2.08	[1.01; 4.28]	1.07	[0.68; 1.69]
Q3	1.34	[0.40; 4.46]	0.72	[0.26; 2.03]	1.62	[0.70; 3.72]	1.13	[0.64; 2.00]
Q4	1.59	[0.41; 6.08]	0.39	[0.11; 1.43]	3.16	[1.19; 8.43]	0.71	[0.39; 1.31]

Model 1: age (years, continuous) and energy intake (kcal/d, continuous). Model 4: Model 1 + physical activity (Met-h/week, continuous), smoking (never, former, and current) and sleep time (hours/day, continuous) sodium (mg/d, continuous)
